# Supplementary material for: Patterns of Intron Gain and Loss in Fungi
Source: PLoS Biol. 2004 Nov 30;2(12):e422. doi: 10.1371/journal.pbio.0020422 (PMC532390; doi:10.1371/journal.pbio.0020422)
Supplement: Table S1 — Also available at http://genes.mit.edu/NielsenEtAl/. (4.3 MB ZIP). [file pbio.0020422.st001.zip › NielsenEtAl/html/992.html]

AN3947.1.NCU10044.1.MG10609.1.FG01348.1


```
 CLUSTAL W (1.82) Multiple Sequence Alignments - Introns Inserted


Sequence 1: MG10609.1	478 aa
Sequence 2: FG01348.1	456 aa
Sequence 3: NCU10044.1	470 aa
Sequence 4: AN3947.1	512 aa
Alignment Length: 546 aa
Number Identitical Residues: 138 aa
Alignment Score (without introns) 7883


MG10609.1 	MAPRQSARRKAAAATTEPKVEIKEVEQETKVVVAQTNGKKSRTIATTVVKKETKVQVKRK
NCU10044.1	MCPSRPTR----SAKSRAATKISSATIEKSRVEETDVDVKKGTRAKGGVKKTEVVEGKVV
FG01348.1 	-MVRTSLR-------NKQTVNYNEDHNDDKMPKAKVAAR-AITTASKIVENANGVASKSV
AN3947.1  	MPPRGARKAAGIETNKRELGDDHELLSTRRQSKRIKSAEQLRSTPSSPQPKSRRVQVKRE
          	     . :  .  :...   .  .               .  : ..    :   *  *  

MG10609.1 	AE--DEDEDEAELSDHCKKEKA~KEEKVELQDIPEIKPAAKRQKTTKGKDQDTMPLAERT
NCU10044.1	VK--KVAARE-------RKGIK1EAKKP---------APKKRK--TKASEEDATPLATRT
FG01348.1 	AA--KAITK--------RKAEP~EAEPENAAPAPAVKAAKKRK--TKAKDEDATPLADRT
AN3947.1  	ARYLQEETTKAENPTLLNTEEL~TEVKSEVSVKVNEEEIKEQTSATKATRKRRSTKKEEA
          	.   .    .:. .   ..         .       .   :: .:**.. :   .   .:

MG10609.1 	VVSTLKR-----AMYIGAHVSAAGG1VQNSVENAAQIG~ANAFALFLKSQRKWANPPLAP
NCU10044.1	PVESMKR-----KLYIGAHVSSAGG1VQNSITNALHIG~ANAFAVFLKSQRKWVSPPLDR
FG01348.1 	AISTLKP-----AMHIGAHVSAAGG1VQNSVTNAIHIG~ANAFALFLKSQRKWANPPLDS
AN3947.1  	EMTPLRVRTQGLRMFVGAHVSAAGG1VFNAVNNSKHVG2GNAFALFLKSQRKWDNPPLQD
          	 : .::  :..  :.:*****:*** * *:: *: ::* .****:******** .***  

MG10609.1 	DARDQFHSSCTKHSYDAAK~HCLPHGSYLVNLAQPEKEKADQAYSNFVDDLKRCDELGIR
NCU10044.1	SAAADFSRLASLHGYSPSL~HVLPHGSYLVNLAQVDKAKADQAYGNFVDDLQRCATLGIK
FG01348.1 	EAKNQFISLCKDHSYSANE~HALPHGSYLVNLAQADEDKANQAYTSFVDDLQRCEQLGIR
AN3947.1  	EHRDNFVKLCKEHDYDAAK2QQLPH-SFTANASS--------------------------
          	.   :*   .. *.*..   : *** *: .* :.                          

MG10609.1 	LYNFHPGSALKDTKEAAIGRIAAQLNRSHKETKS--VITVVENMAGSGNVVGSTFEDLRD
NCU10044.1	LYNFHPGSTGGEPIEEACARIASQLNRAHKEKGTGEVVTVIENMCGSGNVIGSRFEDLKM
FG01348.1 	LYNFHPGSTGGDARPAAIGRIAAQLNKSHKATKT--VVTVLENMAGSGNVIGSTWEDLRD
AN3947.1  	-----PGSTNKTPLSSALARLAKALTNALAATSN--VVPVLETMCGHGSTIGGSLSEFRD
          	     ***:   .   * .*:*  *..:   . .  *:.*:*.*.* *..:*.  .::: 

MG10609.1 	IIELV--EDKSRVGVCIDTCHAFAAGYDLRTPEAFKKTFDEFDRIVGNKYLKAFHL~NDS
NCU10044.1	IIDGV--EDKSRVGVCIDTCHAFAAGHDLRTPEAFEKTMKRFDEIVGLKYLKAFHL~NDS
FG01348.1 	IIELV--EDKSRVGVCIDTCHAFAAGLDLRTPEAFKKTFDSFNEIVGHEYLKAFHL1NDS
AN3947.1  	LLALIPKEYHSRIGVCIDTCHSFAAGYDLVSPAGFQAFLKEFEDLIGIQHLRALHL~NDS
          	::  :..* :**:********:**** ** :* .*:  :. *: ::* ::*:*:** ***

MG10609.1 	KAPFGSNRDLHANIGTGFLGLRAFHSVVNHAPFQDLPMVLETPID---------------
NCU10044.1	KAPFGSCRDLHANIGTGFLGLRAFHNLMNYEAVQGLPMVLETPIE---------------
FG01348.1 	KAPFNSKRDLHANIGTGFLGLRAFHSIMNHAPFAGMPMVLETPID---------------
AN3947.1  	KAPGGSKRDLHANIGTGFLGLRAFHNVMNEPRFEGLPMILETPIDRIPAAATNRAKQEAA
          	*** .* ******************.::*   . .:**:*****:  .::::. :...::

MG10609.1 	----------------------KKDASGKTVEDRQVWADEIKLLESLIEMDAESDEFQKL
NCU10044.1	----------------------EKGADGKMVENKQIWADEIKLLESLVGMDAESEDFKKM
FG01348.1 	----------------------RPGADGKSVEDKKVWADEIKLLERLVGMDAESDEFKEL
AN3947.1  	EEDVESGASDDEIKPKTKKKQQKKPAAAKAVPDYSIWAREIALLESLIGMDPESDEFRAL
          	... .:.::... ...:......  * .* * : .:** ** *** *: **.**::*: :

MG10609.1 	AEKLQKSGAAERKRVQEQVDKKAEKDAKKAAPKGKA--------------VKGRKKK---
NCU10044.1	EEELQKRGASERAKIQEQVDKKAEKDAKKGAGKGKKGAAAAGGSISSF--FKRKVKE---
FG01348.1 	EIELQSKGESERNKIQDQVDRKLAQDAKKGTKKG----------------GKKKKKK---
AN3947.1  	EAELSEEGRETREKHMEQYLRKQETEEKKKAKSGGKQKTLIGMMNGSKGRGKSTTKKGYE
          	  :*.. *   * :  :*  :*   : ** : .*    :  .   .: .  *   *:. .

MG10609.1 	-AETDEDSE--
NCU10044.1	-ESEGEDHE--
FG01348.1 	-DESDE-SE--
AN3947.1  	TESEDEGCQSC
          	: . .*. ::.
```
